# MR 4D flow-based mean pulmonary arterial pressure tracking in pulmonary hypertension

**DOI:** 10.1007/s00330-020-07287-6

**Published:** 2020-09-24

**Authors:** Ursula Reiter, Gabor Kovacs, Clemens Reiter, Corina Kräuter, Volha Nizhnikava, Michael Fuchsjäger, Horst Olschewski, Gert Reiter

**Affiliations:** 1 Division of General Radiology, Department of Radiology, Medical University of Graz, Graz, Austria; 2 Division of Pulmonology, Department of Internal Medicine, Medical University of Graz, Graz, Austria; 3 Ludwig Boltzmann Institute for Lung Vascular Research Graz, Graz, Austria; 4 Institute of Medical Engineering, Graz University of Technology, Graz, Austria; 5 Research & Development, Siemens Healthcare Diagnostics GmbH, Graz, Austria

**Keywords:** Pulmonary hypertension, Hemodynamics, Magnetic resonance imaging, Follow-up studies

## Abstract

**Objectives:**

Longitudinal hemodynamic follow-up is important in the management of pulmonary hypertension (PH). This study aimed to evaluate the potential of MR 4-dimensional (4D) flow imaging to predict changes in the mean pulmonary arterial pressure (mPAP) during serial investigations.

**Methods:**

Forty-four adult patients with PH or at risk of developing PH repeatedly underwent routine right heart catheterization (RHC) and near-term MR 4D flow imaging of the main pulmonary artery. The duration of vortical blood flow along the main pulmonary artery was evaluated from MR 4D velocity fields using prototype software and converted to an MR 4D flow imaging-based mPAP estimate (mPAP_MR_) by a previously established model. The relationship of differences between RHC-derived baseline and follow-up mPAP values (ΔmPAP) to corresponding differences in mPAP_MR_ (ΔmPAP_MR_) was analyzed by means of regression and Bland-Altman analysis; the diagnostic performance of ΔmPAP_MR_ in predicting mPAP increases or decreases was investigated by ROC analysis.

**Results:**

Areas under the curve for the prediction of mPAP increases and decreases were 0.92 and 0.93, respectively. With the natural cutoff ΔmPAP_MR_ = 0 mmHg, mPAP increases (decreases) were predicted with an accuracy, sensitivity, and specificity of 91% (91%), 85% (89%), and 94% (92%), respectively. For patients in whom 4D flow allowed a point estimate of mPAP (mPAP > 16 mmHg), ΔmPAP_MR_ correlated strongly with ΔmPAP (*r* = 0.91) and estimated ΔmPAP bias-free with a standard deviation of 5.1 mmHg.

**Conclusions:**

MR 4D flow imaging allows accurate non-invasive prediction and quantification of mPAP changes in adult patients with PH or at risk of developing PH.

**Trial registration:**

ClinicalTrials.gov identifier: NCT00575692 and NCT01725763

**Key Points:**

*• MR 4D flow imaging allows accurate non-invasive prediction of mean pulmonary arterial pressure increases and decreases in adult patients with or at risk of developing pulmonary hypertension.*

*• In adult patients with mean pulmonary arterial pressure > 16 mmHg, MR 4D flow imaging allows estimation of longitudinal mean pulmonary arterial pressure changes without bias with a standard deviation of 5.1 mmHg.*

## Introduction

Pulmonary hypertension (PH) is a progressive, heterogeneous, potentially life-shortening pathophysiological condition diagnosed invasively by right heart catheterization (RHC) as a mean pulmonary arterial pressure (mPAP) exceeding 20 mmHg at rest [1]. The current guidelines recommend a multidimensional approach for PH treatment monitoring, including the evaluation of clinical, cardiopulmonary exercise, hematologic, and imaging parameters [2–6]. Serial assessment of the hemodynamic profile by RHC is recommended to guide therapy decisions particularly in case of clinical deterioration [2, 3].

Even though the role of cardiac MRI in the clinical management of PH is not yet established, the method offers various tools particularly suitable for PH treatment monitoring and patient follow-up. Allowing for an accurate evaluation of cardiac function and morphology, various quantitative MRI parameters have been reported to be significantly altered in PH [7–20] including, among others, the appearance of vortical blood flow along the main pulmonary artery as assessed by time-resolved, three-directional MR phase contrast (4D flow) imaging [19, 21–28]. Moreover, the duration of vortical blood flow was introduced as a potential 4D flow metric for accurate non-invasive estimation of elevated mPAP [26, 27], making MR 4D flow imaging particularly interesting for non-invasive mPAP tracking. Although there are sparse reports of cases featuring changes in the appearance of vortical blood flow patterns in chronic thromboembolic PH after percutaneous transluminal pulmonary angioplasty [29] and pulmonary thromboendarterectomy [30], it remains unknown whether the observed pressure relationship of vortical blood flow in the main pulmonary artery persists during PH therapy and whether differences in the duration of vortical blood flow along the main pulmonary artery can predict mPAP changes during treatment.

The purpose of the present study was therefore to compare MR 4D flow imaging and RHC-derived mPAP differences from serial investigations of patients with PH or at risk of developing PH and to analyze the potential of MR 4D flow to non-invasively predict mPAP changes during the clinical workup of such patients.

## Materials and methods

### Study population

Forty-four subjects with PH or at risk of developing PH (including patients with connective tissue diseases, interstitial lung disease, and portal hypertension), who underwent baseline and follow-up RHC and MR 4D flow imaging at 1.5 T or 3 T between August 2006 and November 2016, were retrospectively enrolled from two prospective studies in which consecutive patients scheduled for routine RHC were also scheduled for near-term comprehensive cardiac MRI (ClinicalTrials.gov identifier NCT00575692 and NCT01725763). The studies complied with the Declaration of Helsinki and were approved by the local ethics review board. Written informed consent was obtained from all participants. Baseline RHC and corresponding cardiac MRI were performed within 8 ± 13 days of each other. Follow-up RHC and cardiac MRI were performed within 8 ± 21 days of each other, 824 ± 839 days from the baseline investigation. No clinically relevant changes in drug treatment or disease state occurred between RHC and the corresponding cardiac MRI.

### Right heart catheterization

RHC was performed with a 7-French quadruple lumen, balloon-tipped, flow-directed Swan-Ganz catheter (Baxter Healthcare Corp.) using the transjugular approach with the patient positioned supine. RHC parameters assessed included mPAP to diagnose PH, as well as the mean pulmonary artery wedge pressure (PAWP), cardiac output (CO), and the pulmonary vascular resistance (PVR), which were used for the clinical classification of PH patients.

### Cardiac magnetic resonance imaging

Cardiac MRI was performed at 1.5 T (Magnetom Sonata, Siemens Healthcare) or 3 T (Magnetom Trio or Magnetom Skyra, Siemens Healthcare) with the patient in the supine position using phased-array body and spine matrix coils. In detail, for 19 patients, baseline and follow-up MRI investigations were performed at 1.5 T; for 16 patients, baseline and follow-up MRI investigations were performed at 3 T; and for 9 patients, baseline investigations at 1.5 T were followed-up by 3 T MRI.

The cardiac MRI protocol included assessment of retrospectively electrocardiographically gated (ECG-gated), two-dimensional, segmented or ECG-gated, real-time cine balanced steady-state free precession (bSSFP) imaging, covering the entire left (LV) and right (RV) ventricles by a stack of contiguous slices in short-axis orientation. 4D flow data were acquired during free breathing in right ventricular outflow tract (RVOT) orientation, covering the main pulmonary artery with gapless slices of a retrospectively ECG-gated, two-dimensional, segmented, spoiled gradient-echo-based cine phase contrast sequence with three-directional velocity encoding. Velocity encoding was set to 90 cm/s in all directions and adapted if necessary to prevent aliasing. Table 1 provides a summary of 1.5T and 3T cine bSSFP and 4D flow protocol parameters.

**Table 1:** Sequence parameters of the cine bSSFP and 4D flow imaging protocols at 1.5 T and 3 T. Typical scan times for cine bSSFP protocols are given for the stack of contiguous slices in short-axis orientation; the typical scan times for 4D flow protocols are given for the stack of slices covering the main pulmonary artery. *GRAPPA*, generalized auto calibrating partial parallel acquisition; *Not applicable due to prospective ECG-gating

Parameters	Cine bSSFP		4D flow	
	Segmented @1.5 T	Real time @3 T	@1.5 T	@3 T
Measured in-plane resolution (mm^2^)	2.2 × 1.4	3.9–4.5 × 2.5–2.8	2.4 × 1.8	2.4 × 1.8
Slice thickness (mm)	8.0	8.0	6.0	4.0
Echo time (ms)	1.2	1.0–1.1	4.1	3.1
Echo spacing (ms)	2.8	2.3–2.5	7.4	5.2–5.9
Flip angle (°)	60	35–60	15	12–15
Measured temporal resolution (ms)	48–54	34–52	89	42–47
Number of reconstructed cardiac phases	30	–*	20	25–30
Bandwidth (Hz/pixel)	930	1240–1502	455	606
Parallel acquisition factor (using GRAPPA)	–	3	2	2
Averaging	1	1	3	2
Imaging time per slice (heartbeats)	7–8	1	57–78	40–60
Typical scan time (min)	4	< 1	8	8

### Image analysis

LV and RV volumetric function parameters were evaluated from manual segmentation of end-diastolic and end-systolic LV/RV endocardial and epicardial borders in short-axis images using the Argus software (Siemens Healthcare). Myocardial trabeculations and papillary muscles were excluded from the LV and RV cavities. Volumetric function parameters assessed included end-diastolic (EDV) and end-systolic (ESV) volumes, stroke volume (SV), ejection fraction (EF), and LV/RV myocardial mass (LVM, RVM).

**Fig. 1:**
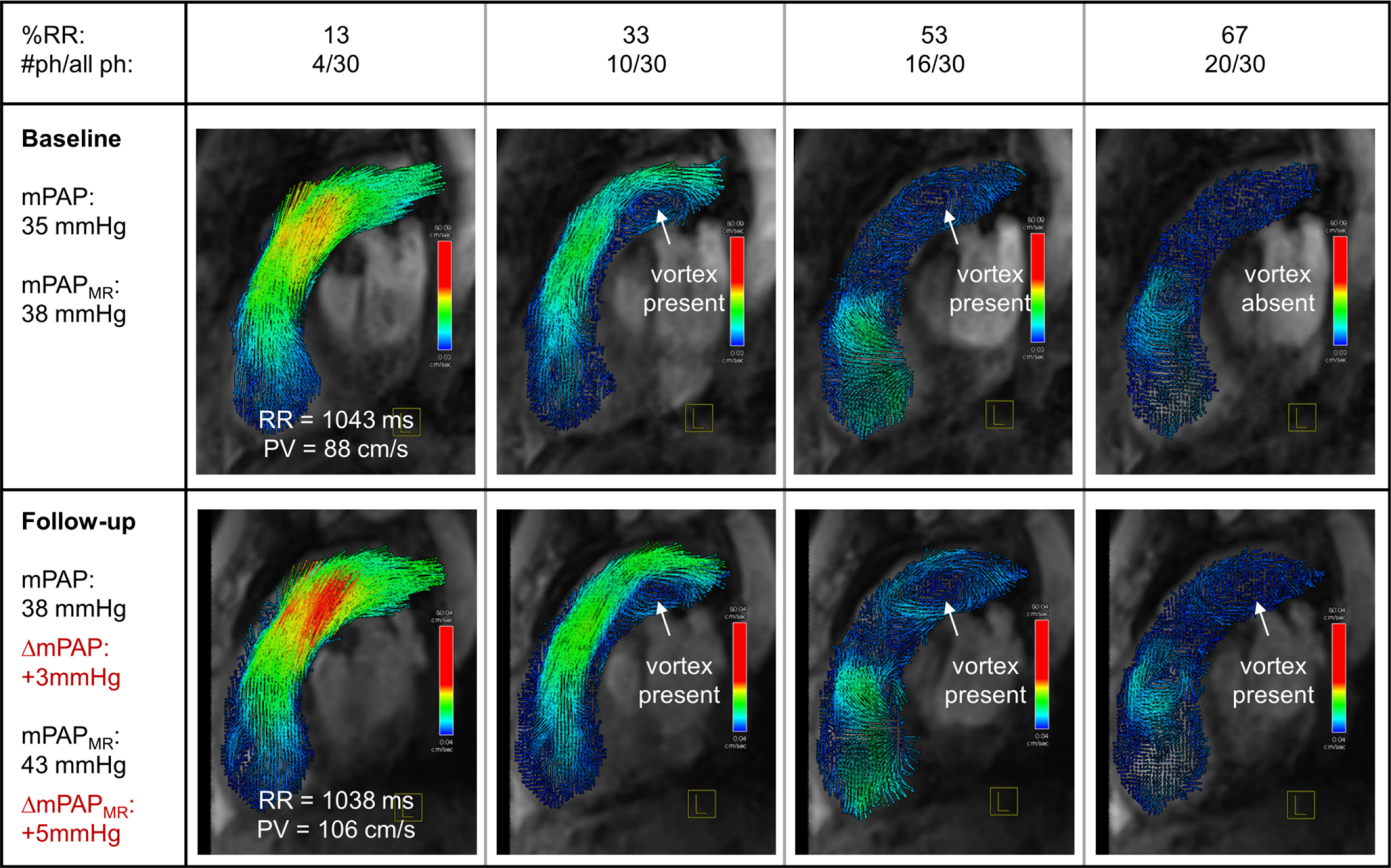
Velocity color-encoded 3-dimensional vector representation of vortical blood flow along the main pulmonary artery in a patient with PH at baseline (upper panel) and 2-year follow-up (lower panel). Color encoding of both representations was set to an equal scale. %RR, percentage of the cardiac interval; #ph, cardiac phase in the RR interval; (Δ)mPAP, mean pulmonary arterial pressure (difference) measured by right heart catheterization; (Δ)mPAP_MR_, mean pulmonary arterial pressure (difference) estimated by 4D flow; PV, main pulmonary artery peak velocity magnitude. mPAP differences are written in red

Baseline and follow-up 4D flow data of the main pulmonary artery were jointly evaluated using a prototype software (4D Flow, Siemens Healthcare) [28, 31]. After cropping spatially aliased structures, background phase correction was applied to all datasets. Blood flow patterns in the main pulmonary artery were analyzed with respect to the existence of vortical blood flow in consensus by two experienced readers (18 years of experience) using multi-planar reconstructed 3-dimensional vector fields in RVOT orientation (Fig. 1) and streamline representations. The duration of vortical blood flow along the main pulmonary artery (t_vortex_) was defined as the percentage of cardiac phases with vortical blood flow in the cardiac interval as previously described [27, 28]. 4D flow-based mPAP (mPAP_MR_) was calculated from t_vortex_ using the piecewise linear model previously given in Reiter et al [27]:

1 $$ {\mathrm{mPAP}}_{\mathrm{MR}}\ \left(\mathrm{in}\ \mathrm{mmHg}\right)\kern1em {\displaystyle \begin{array}{cc}\le 16\kern5.5em &\ \mathrm{for}\ {\mathrm{t}}_{\mathrm{vortex}}=0\\ {}=16+0.63\cdotp {\mathrm{t}}_{\mathrm{vortex}}&\ \mathrm{for}\ {\mathrm{t}}_{\mathrm{vortex}}>0\end{array}} $$

Changes in mPAP and mPAP_MR_ were calculated as the differences between follow-up and baseline values and are denoted by ΔmPAP and ΔmPAP_MR_. An increase (decrease) in mPAP was defined as ΔmPAP > 0 (ΔmPAP < 0) together with the condition mPAP > 16 mmHg at follow-up (mPAP > 16 mmHg at baseline) to enable the comparison with respective increases (decreases) in ΔmPAP_MR_ in the entire study population, where, due to the absence of vortical blood flow for mPAP ≤ 16 mmHg, t_vortex_ = 0 allows only an interval-estimate of ΔmPAP_MR_.

### Statistical analysis

Mean values are given together with standard deviations; sensitivities and specificities are specified together with 95% confidence intervals in parentheses. Statistical analysis was performed using NCSS (Hintze, J. (2008) NCSS, LLC.). For statistical tests, a significance level of 0.05 was employed.

Differences in mean parameter changes from zero between baseline and follow-up investigations were analyzed by one-sample *t* test; relationships of parameter changes to ΔmPAP were analyzed by correlation analysis. The relationships between mPAP and mPAP_MR_ as well as ΔmPAP and ΔmPAP_MR_ were investigated by means of regression and Bland-Altman analysis. Dependencies of biases on MR field strength were analyzed by unpaired *t* test or analysis of variance (ANOVA) of three groups (baseline and follow-up at 1.5 T, baseline and follow-up at 3 T, baseline at 1.5 T and follow-up at 3 T). The diagnostic performance of mPAP_MR_ in predicting PH and the diagnostic performance of ΔmPAP_MR_ in predicting increases or decreases of mPAP were analyzed by receiver operating characteristic (ROC) curve analysis. Optimal cutoffs were defined as those that maximized the sum of sensitivity and specificity, whereas mPAP_MR_ = 20 mmHg for diagnosing PH and ΔmPAP_MR_ = 0 mmHg for diagnosing increases or decreases of mPAP were termed “natural cutoffs” due to the analogy to mPAP-definitions in RHC.

## Results

### Study population

**Fig. 2:**
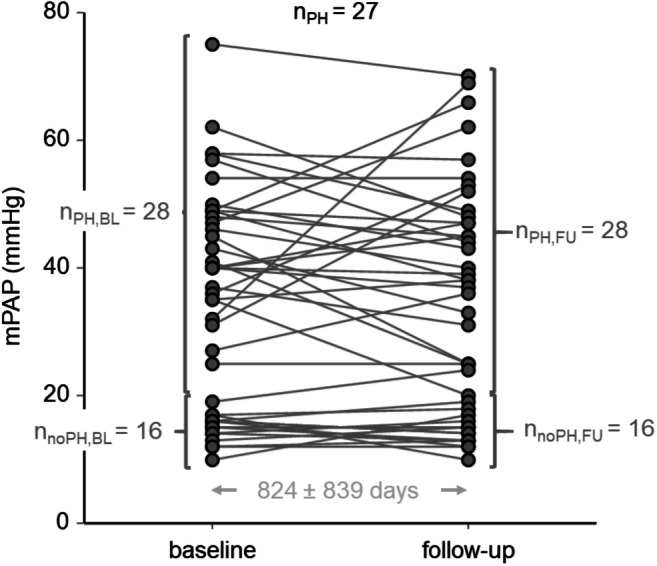
Distributions of mPAP at baseline and at follow-up as well as the mPAP changes of the study population. n_PH,BL_, number of subjects with PH at baseline; n_noPH,BL_, number of subjects without PH at baseline; n_PH,FU_, number of subjects with PH at follow-up; n_noPH,FU_, number of subjects without PH at follow-up; n_PH_, number of subjects with PH at baseline and follow-up

At baseline, PH was diagnosed by RHC in 28 of 44 patients: 16 subjects were classified as having PH due to pulmonary arterial hypertension (PAH), 2 subjects as having PH due to lung diseases, 7 subjects as having chronic thromboembolic PH (CTEPH), and 3 subjects as having PH secondary to multifactorial mechanisms. At follow-up, one patient without PH at baseline developed PAH, and one patient with CTEPH at baseline revealed normal mPAP after surgical treatment. Consequently, PH was diagnosed in 28 patients at follow-up. mPAP > 16 mmHg was found in 31 patients at baseline, in 32 patients at follow-up, and mPAP > 16 mmHg was present in 30 patients at baseline and follow-up. Figure 2 illustrates the distributions of mPAP at baseline and at follow-up as well as the mPAP changes of the study population. The demographic and hemodynamic characterization of the study population is summarized in Table 2 together with LV and RV volumetric function parameters at baseline and follow-up.

**Table 2:** Summary of demographic, right heart catheterization (RHC), and cardiac MR imaging parameters of the study population at baseline and follow-up, together with differences in parameters between baseline and follow-up, and the correlations of differences with mPAP changes (r_ΔmPAP_). Significant differences from 0 are indicated by *, significant correlations by ^+^. *n*, number of subjects at baseline and follow-up; *n*_*PH*_, number of subjects with PH at baseline and follow-up; *n*_*non-PH*_, number of subjects without PH at baseline and follow-up

Parameter	All (*n* = 44)				PH (*n*_PH_ = 27)				Non-PH (*n*_non-PH_ = 15)		
	Base	FU	Diff	r_ΔmPAP_	Base	FU	Diff	r_ΔmPAP_	Base	FU	Diff
F/M (nr)	26/18	26/18			12/15	12/15			13/2	13/2	
Age (years)	59 ± 13	61 ± 13	2 ± 2*		60 ± 14	63 ± 14	2 ± 2*		57 ± 11	59 ± 11	2 ± 2*
Height (cm)	169 ± 9	169 ± 9	0 ± 3		171 ± 8	170 ± 9	0 ± 2		166 ± 10	166 ± 10	0 ± 3
Weight (kg)	76 ± 13	76 ± 13	0 ± 6		78 ± 12	79 ± 12	1 ± 6		71 ± 15	72 ± 15	0 ± 2
RHC											
mPAP (mmHg)	34 ± 17	34 ± 18	0 ± 10		45 ± 11	45 ± 13	0 ± 13		15 ± 2	14 ± 2	0 ± 3
PAWP (mmHg)	8 ± 3	9 ± 4	0 ± 4	0.31	9 ± 3	9 ± 5	0 ± 5	0.28	7 ± 2	7 ± 2	0 ± 3
PVR (WU)	6.1 ± 4.4	5.4 ± 4.3	- 0.7 ± 2.8	0.75^+^	8.7 ± 3.4	7.7 ± 3.4	- 1.0 ± 3.4	0.77^+^	1.5 ± 0.8	1.5 ± 0.5	0.0 ± 0.6
CO (l/min)	4.9 ± 1.8	5.1 ± 1.3	0.1 ± 1.6	- 0.07	4.4 ± 1.1	5.0 ± 1.3	0.7 ± 1.0*	- 0.16	6.1 ± 2.3	5.3 ± 1.1	- 0.8 ± 2.2
Cardiac MR imaging											
LVEDV (ml)	120 ± 33	124 ± 36	4 ± 30	- 0.19	122 ± 40	127 ± 42	5 ± 35	- 0.34	113 ± 17	118 ± 20	5 ± 21
LVESV (ml)	42 ± 23	43 ± 20	0 ± 18	0.01	45 ± 29	44 ± 25	-1 ± 20	- 0.05	37 ± 9	41 ± 11	4 ± 14
LVSV (ml)	78 ± 21	81 ± 24	3 ± 20	- 0.30^+^	77 ± 25	83 ± 28	6 ± 23	- 0.47^+^	77 ± 14	77 ± 12	0 ± 11
LVEF (%)	66 ± 11	66 ± 10	1 ± 9	- 0.17	64 ± 13	67 ± 12	2 ± 9	- 0.22	68 ± 5	66 ± 5	- 2 ± 8
LVM (g)	99 ± 26	104 ± 29	5 ± 23	- 0.09	102 ± 27	110 ± 29	8 ± 20*	- 0.09	96 ± 26	96 ± 27	0 ± 29
RVEDV (ml)	183 ± 88	194 ± 93	- 11 ± 54	0.32^+^	214 ± 88	236 ± 96	22 ± 49*	0.22	119 ± 22	125 ± 24	6 ± 24
RVESV (ml)	112 ± 81	111 ± 80	- 1 ± 38	0.61^+^	140 ± 86	140 ± 90	0 ± 37	0.67^+^	54 ± 14	62 ± 19	8 ± 22
RVSV (ml)	71 ± 22	84 ± 32	12 ± 33*	- 0.19	74 ± 23	96 ± 34	22 ± 34*	- 0.40^+^	64 ± 17	63 ± 12	- 2 ± 18
RVEF (%)	43 ± 15	47 ± 14	4 ± 14	- 0.45^+^	38 ± 15	44 ± 15	6 ± 13*	- 0.69^+^	54 ± 9	51 ± 9	- 2 ± 14
RVM (g)	80 ± 49	84 ± 49	5 ± 24	0.31^+^	99 ± 50	109 ± 47	10 ± 25*	0.23	43 ± 11	43 ± 9	0 ± 8

### MR 4D flow-based prediction of PH and mPAP increase/decrease

Using mPAP_MR_ to predict PH, the area under the curve (AUC) was 1.00 at both baseline and follow-up. At baseline, optimal and natural cutoff coincided, such that the cutoff of mPAP_MR_ = 20 mmHg resulted in an accuracy of 100% (92–100%), a sensitivity of 100% (88–100%), and a specificity of 100% (79–100%) for diagnosing PH. At follow-up, the optimal (23 mmHg) and natural cutoffs did not coincide; the cutoff of mPAP_MR_ = 20 mmHg resulted in an accuracy of 95% (84–99%), a sensitivity of 100% (88–100%), and a specificity of 88% (62–98%) for diagnosing PH.

**Fig. 3:**
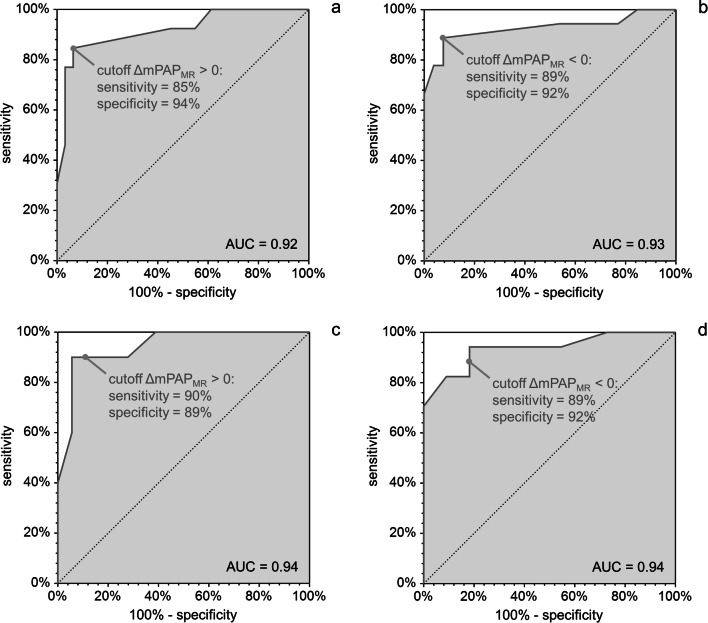
ROC curves for the prediction of mPAP increase (**a**) and decrease (**b**) based on ΔmPAP_MR_ for the entire study population, as well as for mPAP increase (**c**) and decrease (**d**) for patients with PH at baseline RHC

AUCs for the prediction of increase or decrease of mPAP between baseline and follow-up based on ΔmPAP_MR_ were 0.92 (0.74–0.96) and 0.93 (0.75–0.98), respectively (Fig. 3a, b). In both cases, the optimal and natural (ΔmPAP_MR_ = 0 mmHg) cutoffs coincided. The accuracy, sensitivity, and specificity for predicting mPAP increase were 91% (78–97%), 85% (56–98%), and 94% (79–99%), respectively, while the corresponding values for predicting mPAP decrease were 91% (78–97%), 89% (65–99%), and 92% (75–99%), respectively.

Restricting the study population to PH patients, the AUC values for the prediction of increase or decrease of mPAP were 0.94 (0.77–0.99) and 0.94 (0.76–0.98), respectively. The natural cutoff ΔmPAP_MR_ = 0 mmHg resulted in an accuracy of 89% (72–98%), sensitivity of 90% (56–100%), and specificity of 89% (65–99%) for predicting mPAP increase, and an accuracy of 86% (67–96%), sensitivity of 88% (64–99%), and specificity of 82% (48–98%) for predicting mPAP decrease (Fig. 3c, d).

### MR 4D flow-based assessment of mPAP and ΔmPAP

**Fig. 4:**
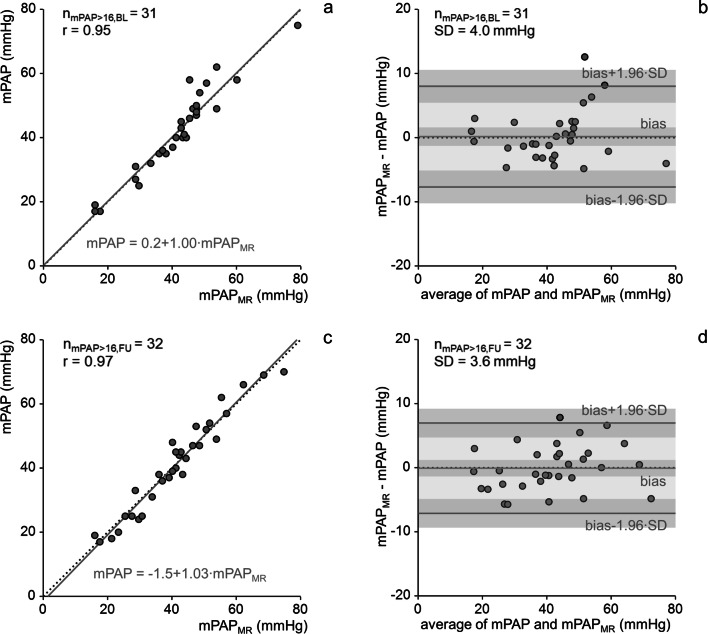
Linear regression and Bland-Altman plots of mean pulmonary arterial pressures measured at right heart catheterization (mPAP) and estimated from vortex duration (mPAP_MR_) at baseline (**a**, **b**) and follow-up (**c**, **d**). Light gray shading indicates the area between 95% limits of agreement, dark gray shadings indicate 95% confidence intervals of bias and 95%-limits of agreement. n_mPAP > 16,BL_, number of subjects with mPAP > 16 mmHg at baseline; n_mPAP > 16,FU_, number of subjects with mPAP > 16 mmHg at follow-up; r, correlation coefficient

The correlation between mPAP and mPAP_MR_ for patients with mPAP > 16 mmHg was strong at both baseline (*r* = 0.95) and follow-up (*r* = 0.97). Bias to RHC as the reference method was not found at either baseline or follow-up (0.2 mmHg, *p* = 0.83 and - 0.1 mmHg, *p* = 0.90 for baseline and follow-up, respectively). Standard deviations of differences were 4.0 mmHg for the baseline and 3.6 mmHg for the follow-up investigation (Fig. 4). Differences between mPAP and mPAP_MR_ did not depend significantly on field strength (*p* = 0.73 for the baseline and *p* = 0.19 for the follow-up investigation).

**Fig. 5:**
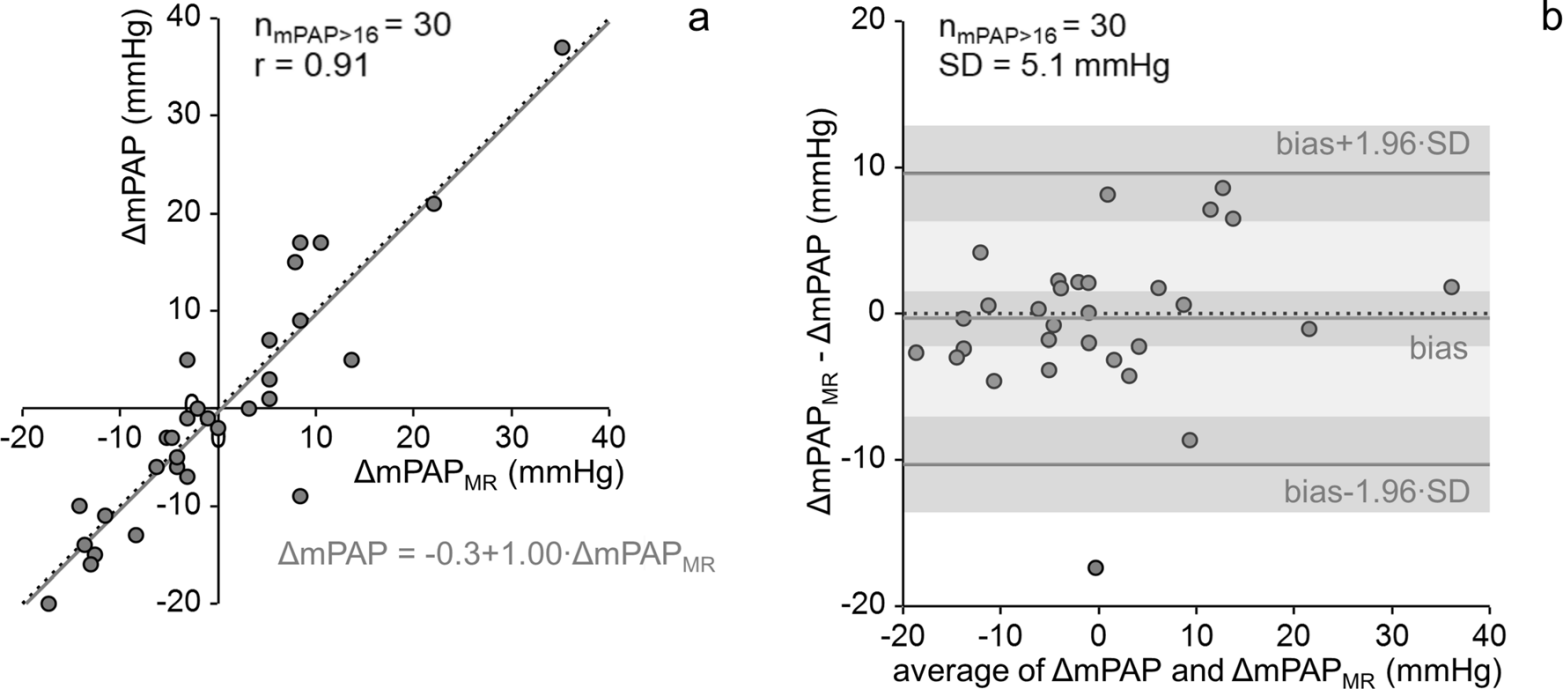
Linear regression (**a**) and Bland-Altman (**b**) plot of mean pulmonary arterial pressure differences between baseline and follow-up measured by right heart catheterization (ΔmPAP). Light gray shading indicates the area between 95% limits of agreement, dark gray shadings indicate 95% confidence intervals of bias and 95% limits of agreement. n_mPAP > 16_, number of subjects with mPAP > 16 mmHg at baseline and follow-up

In patients with mPAP > 16 mmHg at baseline and follow-up, ΔmPAP correlated strongly with ΔmPAP_MR_ (*r* = 0.91). There was no significant bias (- 0.3 mmHg, *p* = 0.71), and the standard deviation of differences was 5.1 mmHg (Fig. 5). The differences between ΔmPAP and ΔmPAP_MR_ did not depend significantly on field strength(s) used at baseline and follow-up (*p* = 0.07).

## Discussion

The most important finding of this study is that MR 4D flow imaging of the main pulmonary artery during PH treatment allows both accurate prediction of the direction of mPAP changes and precise, non-invasive estimation of the magnitude of mPAP changes in patients with mPAP > 16 mmHg.

Stabilization and improvement of pulmonary hemodynamics are major objectives in the management of patients with PH [3, 32]. Doppler echocardiography is currently the primary non-invasive method for the evaluation of RV function and pulmonary arterial pressure during PH therapy [6, 24]. The technique, however, has limitations with respect to reproducibility [5, 33–37] and the accuracy of pulmonary arterial pressure estimation [36–39], potentially limiting the reliability of echocardiography for serial assessments of individual patients. Cardiac MRI has increasingly been used in PH studies as a reference standard for the evaluation of ventricular function [40–43]. These studies have identified progressive RV dilatation and decreased RV EF at follow-up cardiac MRI as important predictors of poor long-term outcome and treatment failure. Moreover, van de Veerdonk et al [41] reported increasing RV volumes and decreased RV EF as early signs of clinical worsening in a group of severe PAH patients, suggesting that RV remodeling may precede disease progression even in patients appearing clinically stable. Notably, significant correlations were also found between RV ESV, RV EF, and RV SV changes and ΔmPAP in the present study.

Even though various routine cardiac MRI parameters and parameter models have been used for the evaluation of mPAP in PH showing their potential of mPAP prediction with standard deviations in the range of 7–10 mmHg [8, 12, 44], cardiac MRI is not yet established as a tool for the non-invasive estimation of mPAP. Reiter et al [27] demonstrated a more accurate 4D flow-based estimation of mPAP > 16 mmHg and high accuracy in the diagnosis of PH from the duration of vortical blood flow in the main pulmonary artery using a threshold of mPAP ≥ 25 mmHg, irrespective of the etiology of PH. Employed with the recently introduced threshold of mPAP > 20 mmHg for the diagnosis of PH [1] in the present study, the technique revealed the accuracy for the non-invasive diagnosis of PH similar to that which it yielded with the former threshold mPAP ≥ 25 mmHg [26–28]. Using a piecewise linear model for non-invasive estimation of mPAP > 16 mmHg [27], mPAP was—in accordance with [27, 28] —predicted without bias and with small standard deviations from RHC at baseline as well as follow-up cardiac MRI. As the differences between mPAP_MR_ and mPAP did not depend on field strength and a main difference in 4D flow protocols at different field strength was the measured temporal resolution, it could be speculated that the measured temporal resolution of the 4D flow acquisition does not play a central role for the accuracy of mPAP estimation.

The standard deviation for the prediction of pressure differences in the present study was lower than anticipated by the law of error propagation, which would result in a standard deviation of $\sqrt{3.6^2+{4.0}^2}=5.4\ \mathrm{mmHg}$. This can be understood to be due to the comparative interpretation of vortical blood flow along the main pulmonary artery observed at baseline and follow-up, which increases the likelihood that a combined over- or underestimation of vortex duration-derived mPAP at baseline and follow-up will be canceled out in the difference (ΔmPAP_MR_). The standard deviation between invasively measured and non-invasively estimated ΔmPAP, however, cannot be expected to be substantially smaller than 5 mmHg, because RHC and MRI measurements were not acquired simultaneously: Assuming a spontaneous mPAP variability of approximately 3 mmHg [26, 45, 46], deviations between RHC and MRI of at least $\sqrt{3^2+{3}^2}=4.2\ \mathrm{mmHg}$ have to be expected, even without taking into account the limited time resolution of vortex duration assessment.

The high accuracy for the prediction of mPAP increases and decreases in PH patients can be interpreted as a natural consequence of the strong correlation between ΔmPAP from RHC and MR. Considering mPAP changes within the range mPAP ≤ 16 mmHg as clinically not relevant (that is—as defined in the present study—no mPAP change), the accuracy of predicted mPAP changes in the whole population was comparable to that in the PH group. This result implies that the absence of vortical blood flow along the main pulmonary artery at baseline and follow-up in patients at risk of developing PH indicates persistent normal mPAP ≤ 16 mmHg [47].

Several limitations of the current study need to be acknowledged. As this was a retrospective analysis of two prospective studies, follow-up of patients was not planned and the follow-up times varied between 1 and 10 years. No patients with PH due to left heart diseases were included in the data analysis, as these patients were not repeatedly followed up by RHC. The study population included patients across a wide mPAP range, which limits the comparability of differences in the diagnosis of PH from the duration of vortical blood flow in the main pulmonary artery based on different mPAP cutoffs for PH. Moreover, only adult patients were recruited. Although protocols used for the assessment of 4D flow at 1.5 T and 3 T MRI differed, the bias of resulting pressure differences to RHC did not differ, indicating that results were not directly affected by the acquisition strategy.

In conclusion, MR 4D flow imaging allows accurate non-invasive prediction and quantification of mPAP changes in adult patients with PH or at risk of developing PH.

## Abbreviations

ΔmPAP: Change in mPAP
ΔmPAP_MR_: Change in mPAP_MR_
4D flow: Time-resolved, three-directional phase contrast imaging
4D: 4-dimensional
ANOVA: Analysis of variance
bSSFP: Balanced steady-state free precession
CTEPH: Chronic thromboembolic PH
ECG-gated: Electrocardiographically gated
EDV: End-diastolic volume
EF: Ejection fraction
ESV: End-systolic volume
GRAPPA: Generalized autocalibrating partial parallel acquisition
LV: Left ventricle
LVM: Left ventricular mass
mPAP: Mean pulmonary arterial pressure
mPAP_MR_: 4D flow-based mPAP estimate
MR: Magnetic resonance
MRI: Magnetic resonance imaging
PAH: Pulmonary arterial hypertension
PAWP: Pulmonary arterial wedge pressure
PH: Pulmonary hypertension
PVR: Pulmonary vascular resistance
RHC: Right heart catheterization
RV: Right ventricle
RVM: Right ventricular mass
RVOT: Right ventricular outflow tract
SV: Stroke volume
t_vortex_: Duration of vortical blood flow along the pulmonary artery
